# Magnetorheological Hybrid Elastomers Based on Silicone Rubber and Magnetorheological Suspensions with Graphene Nanoparticles: Effects of the Magnetic Field on the Relative Dielectric Permittivity and Electric Conductivity

**DOI:** 10.3390/ijms20174201

**Published:** 2019-08-27

**Authors:** Ioan Bica, Octavian Mădălin Bunoiu

**Affiliations:** Department of Physics, West University of Timisoara, 300223 Timisoara, Romania

**Keywords:** magnetodielectric effect, hybrid magnetorheological elastomer, membrane, silicone rubber, graphene nanoparticles, carbonyl iron

## Abstract

Hybrid magnetorheological elastomers (hMREs) were manufactured based on silicone rubber, silicone oil, carbonyl iron microparticles, graphene nanoparticles and cotton fabric. Using the hMREs, flat capacitors (FCs) were made. Using the installation described in this paper, the electrical capacitance and the coefficient of dielectric losses of the hMREs were measured as a function of the intensity of the magnetic field superimposed over an alternating electric field. From the data obtained, the electrical conductivity, the relative dielectric permittivity and magnetodielectric effects are determined. It is observed that the obtained quantities are significantly influenced by the intensity of the magnetic field and the amount of graphene used.

## 1. Introduction

Magnetorheological materials consist of a silicone oil-based matrix, mineral oil, elastomer, etc., in which a magnetisable phase and additives are dispersed. The magnetisable phase is in the form of ferro-ferrimagnetic particles, and the additives are in the form of nano-microparticles. The latter can be either electroconductive or dielectric.

In the literature, these materials are known by the generic name “smart materials” [1,2,3,4,5,6,7,8]. They have a certain peculiarity, which consists in the fact that their physical properties change significantly when applying external factors in the form of magnetic fields, electromagnetic fields, mechanical tensions, etc. [9,10,11,12,13]. This property of magnetorheological materials is used in technical applications [14,15]. Recently, due to the increased scientific and applied interest, the scientific community has attributed great importance to magnetorheological elastomers (MREs). In MREs, the magnetisable microparticles and additives are “frozen” by polymerization into the polymer. In this way, the sedimentation coefficient is much lower than that of the magnetorheological suspensions, giving them stable physical properties over time. In a magnetic field, the magnetisable phase in the elastic matrix of the MREs is oriented along the field lines. Aggregates are created in the form of chains, which have the effect of drastically modifying the rheological properties. These properties are of use in the production of vibration and seismic shock dampers [16], and in the development of sensors for mechanical deformation and stresses [17], actuators [18], etc. In Refs. [19,20,21], it is reported that the electrical conductivity of MREs increases in a magnetic field, which is a useful property in the realization of sensors and actuators [7,9,10].

Recently, academia has been concerned with the study of magnetodielectric materials induced by a magnetic field superimposed on a low-, high- or very high-frequency alternative electric field.

In Ref. [22], the effects induced by magnetic fields in ceramics in a high-frequency electric field are reported. In these materials, the components of the complex dielectric permittivity are noticeably modified in a static magnetic field superimposed over a high frequency electric field. In Refs. [23,24], using magnetic fluids based on Fe nanoparticles, an increase of up to 15% in the magnetodielectric effects was reported through applying a static magnetic field superimposed on an electric field with frequencies of up to 5 MHz. Spectacular increases in magnetodielectric effects were obtained in Ref. [25]. Here, the magnetodielectric effects induced in MREs based on Fe, NdFeB, and Fe_3_O_4_ microparticles were up to 150% higher when superimposing the static magnetic field over a high-frequency electric field. In a recent paper [26], it was reported that magnetodielectric effects are also relevant in magnetorheological biosuspensions. Here, it was mentioned that magnetodielectric effects can be used for biomedical purposes. This assessment was based on the use of thermal transport of bioactive compounds. Following this research direction, in this paper, hybrid MREs are manufactured. They have in their composition a textile fabric, impregnated with a mixture made of silicone rubber, silicone oil, graphene nanoparticles and carbonyl iron microparticles, which polymerize between two conductive plates. Using the installation described in the paper, measurements of electric capacitance and of coefficients of dielectric losses are made in an alternative electric field with a frequency of 1 kHz, superimposed over a static magnetic one. From the obtained data, the relative dielectric permittivity and electrical conductivity of hMREs are determined. It is shown that the determined quantities depend on the intensity of the external magnetic field and on the composition of the hMREs.

## 2. Results and Discussion

**Figure 1:**
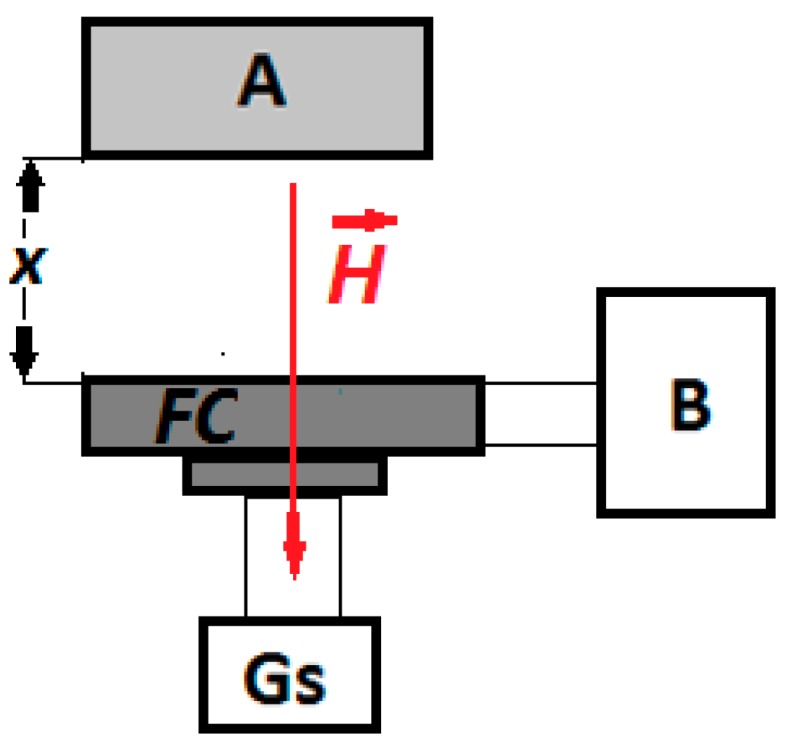
Experimental setup (overall configuration): A—neodymium magnet; B—impedance meter (type ET-20); FC—working flat capacitor; $\vec{H}$—magnetic field strength vector; Gs—Gaussmeter, type DX-102; h—Hall probe; *x*—adjustable distance.

**Figure 2:**
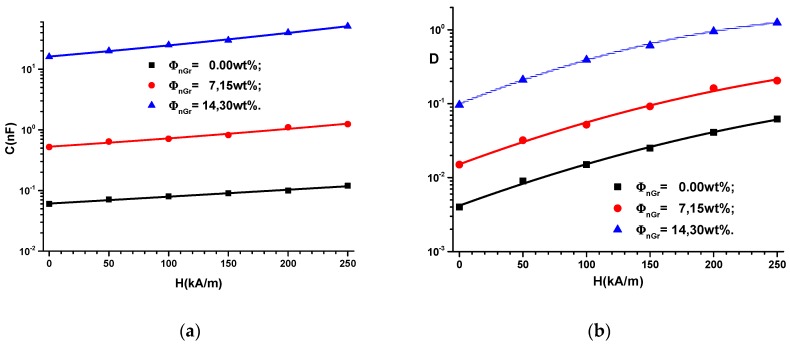
(**a**) The capacitance *C* of the FC; (**b**) the dielectric loss factor *D* dependence on the intensity *H* of the magnetic field and on the mass fractions $\Phi_{nGr}$ of the graphene nanoparticles.

The experimental setup used to measure the capacitance *C* and the dielectric loss coefficient *D* is described in Figure 1. Using the adjustment device, the distances between A and FC are fixed in such a way that the intensity of the magnetic field is increased in steps of 50 kA/m up to values of 250 kA/m. Using the bridge B, we measured the capacitance *C* and the dielectric loss coefficient *D* of the FCs in the electric field with f = 1 kHz frequency at intervals of t = 5 s, starting with the application of the magnetic field. The obtained results are presented in Figure 2.

It can be seen from Figure 2 that the capacitance *C* and the coefficient of dielectric losses *D* of the FCs increase with the increase of the intensity *H* of the external magnetic field. In contrast, for fixed values of *H*, *C* and *D* increase with increasing mass fraction of graphene nanoparticles.

It is known [1,2,3,4,5,6,7,8,9,10,11,12,13,14,15,16,17,18,19,20,21,22,23,24,25,26,27,28,29] that in a magnetic field, the CI microparticles of the hMRE instantaneously become magnetic dipoles. They orient themselves, in time, along the filed lines of the magnetic field. The measurements are performed at fixed intervals of time, and therefore the time-dependence of the measured quantities is not shown in Figure 2.

The capacitance of the FC, neglecting edge effects, can be calculated by:

(1) $$C=\frac{\varepsilon_{0}\varepsilon^{\prime }Ll}{d}$$

where $\varepsilon_{0}$ is the dielectric permittivity of vacuum, $\varepsilon^{\prime }$ is the relative dielectric permittivity, *L* is the length, *l* is the width and *d* is the thickness of the membrane.

For ε0=8.85×10−12Fm, L=50 mm, l=40 mm and d=1.2 mm, in Expression (1), the relative dielectric permittivity can be obtained as follows:

(2) $$\varepsilon^{\prime }=67.56\times C{\left(H\right)}_{\Phi_{nGr}}$$

**Figure 3:**
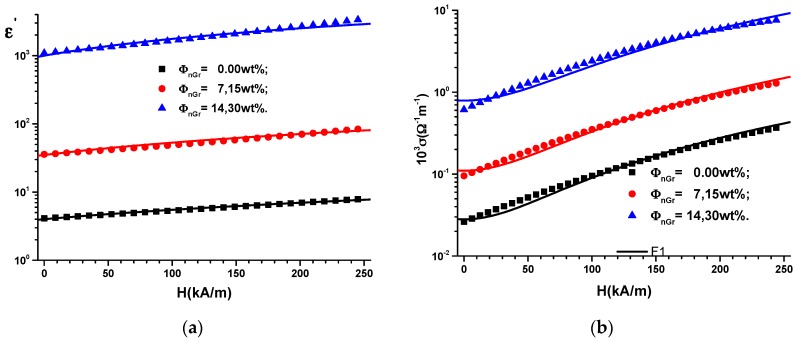
(**a**) Relative dielectric permittivity ε’; (**b**) electrical conductivity σ as function of intensity *H* of the magnetic field and mass fractions $\Phi_{nGr}$ of graphene nanoparticles (dots = experimental data, lines = theoretical result).

The functions $C{\left(H\right)}_{\Phi_{nGr}}$ from Figure 2a are inserted in Expression (2), and in Figure 3a, the variation of $\varepsilon^{\prime }$ with intensity *H* of the static magnetic field superimposed on the alternative electric field is obtained.

Due to interfacial polarization of the nanographene [30], the relative dielectric permittivity of the nMRE (Figure 3a) increases with *H* and is noticeably influenced by the increase in nanographene mass fraction $\Phi_{nGr}$. Variation of the relative dielectric permittivity ε’ with the intensity *H* of the magnetic field for the used quantities of graphene can be approximated, as can be observed in Figure 3a, by linear functions, as:

(3) $$\varepsilon^{\prime }=\varepsilon_{0}^{\prime }+\alpha H=\{\begin{array}{c} 4+0.0148\cdot H\left(\mathrm{kA}/m\right)\\ 35+0.18\cdot H\left(\mathrm{kA}/m\right)\\ 1000+7.8\cdot H\left(\mathrm{kA}/m\right) \end{array}$$

where $\varepsilon_{0}^{\prime }$ is the relative dielectric permittivity of the hMREs at *H* = 0 and α is a physical quantity that depends on the intensity of the magnetic field and the quantity of the graphene in the hMREs.

Corroborating the results in Figure 3a with Relation (3), it can be observed that in the absence of the magnetic field, the relative dielectric permittivity increases by 8.75 times for the hMRE with $\Phi_{nGr}=7.15\;\mathrm{wt}\%$ and by 250 times for the hMRE with $\Phi_{nGr}=14.30\;\mathrm{wt}\%$, compared to the hMRE with $\Phi_{nGr}=0.00\;\mathrm{wt}\%$. In a magnetic field, the slope of the functions of Figure 3a increase by 12 times for hMRE with $\Phi_{nGr}=7.15\;\mathrm{wt}\%$ and by 527 times for the hMRE with $\Phi_{nGr}=14.30\;\mathrm{wt}\%$, compared to the hMRE with no graphene nanoparticle content.

We define the magnitude of the magnetodielectric effect by the expression:

(4) $$MDE\left(\%\right)=\frac{\varepsilon^{\prime }-\varepsilon_{0}^{\prime }}{\varepsilon_{0}^{\prime }}\cdot 100$$

where the notations for permittivity are the same as those previously introduced.

**Figure 4:**
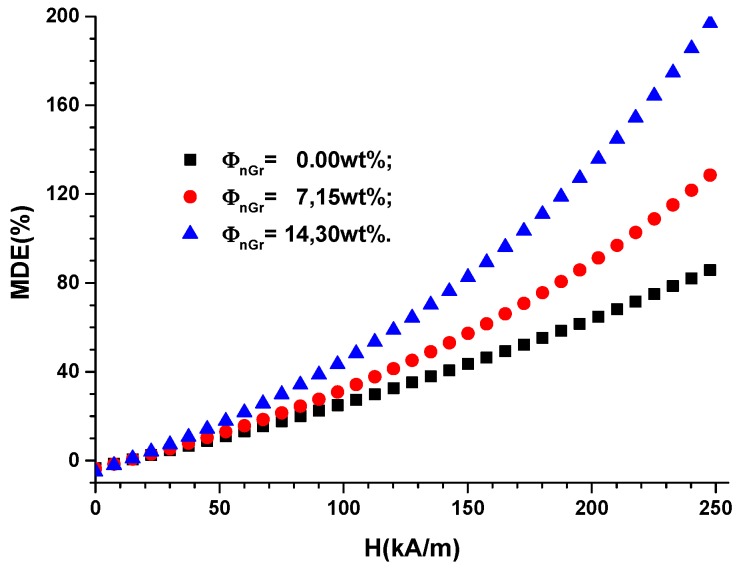
The magnetodielectric effect MDE versus the magnetic field intensity *H* for the mass fractions $\Phi_{nGr}$ of graphene nanoparticles.

In Relation (4), the results of Figure 3a are inserted for the hMREs, and the magnitude of the magnetodielectric effect are obtained in Figure 4.

The relaxation polarization of silicone rubber [27] coupled with the interfacial one of graphene nanoparticles [30] and the magnetoconstriction phenomenon [6,7,8,9,10] have the effect of increasing MDE with *H* and $\Phi_{nGr}$, as shown in Figure 4.

Between the electrical conductivity σ and the dielectric loss coefficient *D*, there is the following relation [28,29]:

(5a) σ=2πfD

where *f* is the frequency, ε_0_ is the dielectric permittivity of the vacuum and *D* the dielectric loss coefficient.

For the frequency *f* = 1000 Hz the Expression (5a) takes the form

(5b) $$10^{3}\sigma \;\left(\Omega^{-1}m^{-1}\right)=6.28\cdot D$$

Using Expression (5b) and the the function $D=D{\left(H\right)}_{\Phi_{nGr}}$ from Figure 2b, we obtain the variation of the electrical conductivity $\sigma =\sigma \;{\left(H\right)}_{\Phi_{nGr}}$ as shown in Figure 3b.

(6) $$10^{3}\cdot \sigma =\sigma_{0}+\beta \cdot H^{2}=\{\begin{array}{c} 28\cdot 10^{-3}+62\cdot 10^{-7}H^{2}\\ 11\cdot 10^{-2}+22\cdot 10^{-6}H^{2}\\ 79\cdot 10^{-2}+13\cdot 10^{-5}H^{2} \end{array}$$

where σ_0_ is the electric conductivity of the hMREs in the absence of the magnetic field and β is the quantity that is measured in $\Omega^{-1}m^{-3}{\mathrm{kA}}^{-2}$.

Based on Figure 3b and Expression (6), it can be observed that by adding graphene nanoparticles, the electron transfer through the SR increases [27,30], which has the effect of increasing the electric conductivity by 3.92 times for the hMRE with $\Phi_{nGr}=7.15\;\mathrm{wt}\%$ and 28.21 times for the hMRE with $\Phi_{nGr}=14.30\;\mathrm{wt}\%$ compared to the hMRE with no graphene nanoparticles.

The parameter β from Expression (6) increased by 3.55 times for the hMRE with $\Phi_{nGr}=7.15\;\mathrm{wt}\%$ and 20.97 times for the hMRE with $\Phi_{nGr}=14.30\;\mathrm{wt}\%$ compared to the hMRE with no graphene content. The increase of parameter β in a magnetic field is due to the constriction effect of the hMREs, as reported in Ref. [27]. On the other hand, the increase of β with the mass fraction $\Phi_{nGr}$ is due to the facilitation of the electron transport by graphene, as shown in Ref. [30].

## 3. Materials and Methods

### 3.1. Materials

The necessary materials for producing hMREs and flat capacitors (FCs) are:

- Silicone Rubber (SR), from RTV-Silicone, a product having a white color, and density 2.30 g/cm^3^;
- Silicone Oil (SO), type C3518 from Sigma-Aldrich, with density 1.08 g/cm^3^;
- Carbonyl Iron (CI), type C3518, from Sigma-Aldrich, in the shape of spherical particles having diameters between 4.5 μm and 5.4 μm, a Fe content of at least 97% and a density of 7.86 g/cm^3^;
- Graphene NanoPowder (nGr), from Sky Spring Nanomaterials Inc., powder with Platelet Nanopowder of thickness between 6 nm and 8 nm, average diameter of 15 µM Graphene and density 2.28 g/cm^3^;
- gauze bandage (FT), from MKD Medicala with a granulation of 30 g/cm^3^;
- six textolite plates (TCu), copper-plated, from Sierra Modellsport.

### 3.2. The Manufacturing of the hMRE Membranes

Stage 1: In a beaker, the mixture of SR with CI and nGr is homogenized for about 30 min, in the proportions specified in Table 1. After 30 min, *S*_1_, *S*_2_ and *S*_3_ samples were obtained, in the form of dark, viscous liquids.

**Figure 5:**
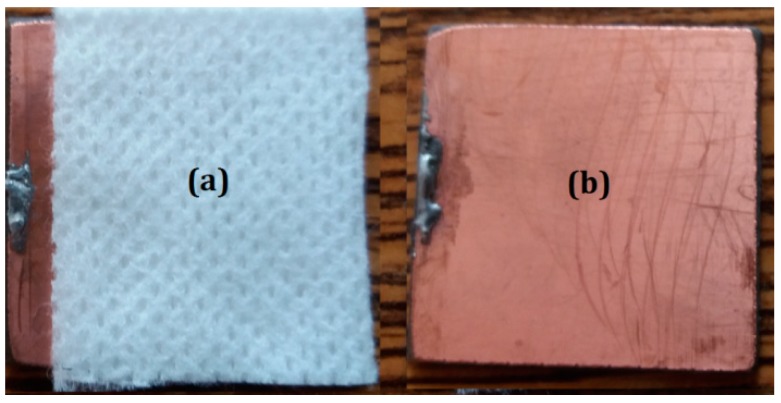
(**a**) Absorbent fabric (FT); (**b**) copper-plated plates (TCu).

Stage 2: The FT, from MKD Medicala (Figure 5a), is reduced to the dimensions of 50 mm × 40 mm. Three packets are prepared from the FT, each having a thickness of 6 mm.

Stage 3: The first batch is impregnated with sample *S*_1_ the second batch with sample *S*_2_ and finally, the third batch with sample *S*_3_. Unpolymerized membranes *M*_1_, *M*_2_ and *M*_3_ are obtained (Table 2).

**Figure 6:**
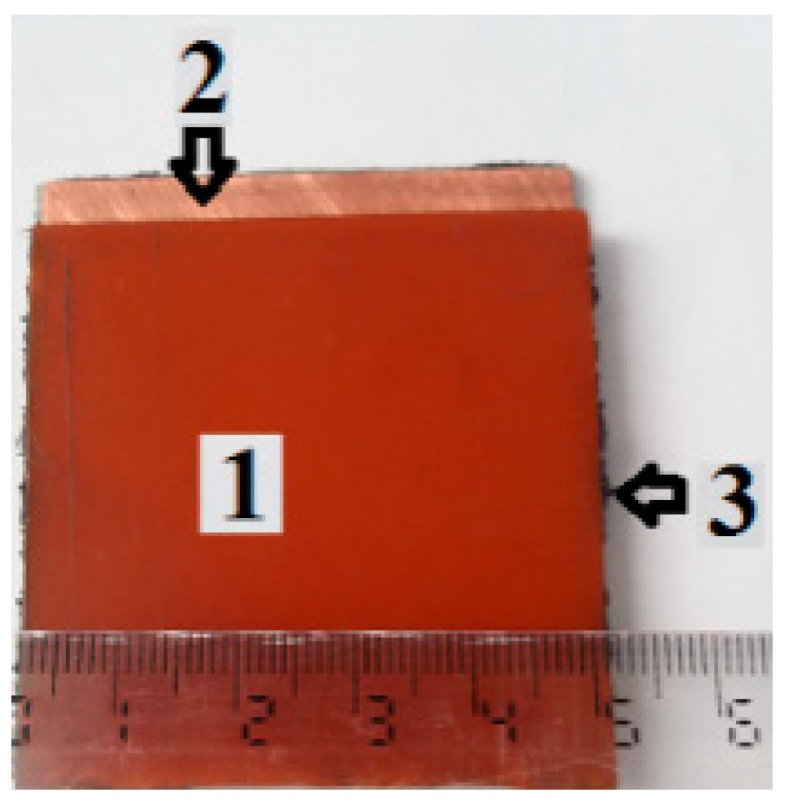
Flat capacitor: 1—TCu plate; 2—copper-plated side of the TCu; 3—hMRE.

**Figure 7:**
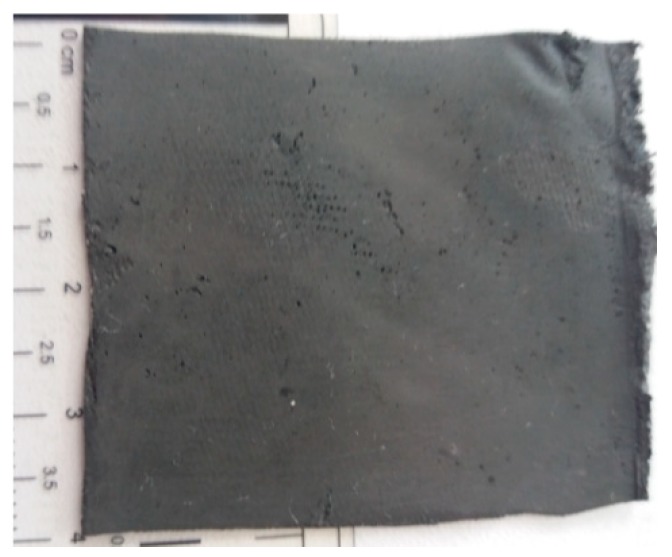
hMRE configuration.

Stage 4: Using the TCu plates, three packets are made, with two plates in each packet. The TCu plates have a square shape with a side of 50 mm. One by one, the membranes *M*_1_, *M*_2_ and *M*_3_ are placed between the TCu plates, with the coppered faces towards the membranes. Each packet is pressed until the distance between the plates is 1.2 mm, after which they are left in air. After approximately 24 h, the membranes *M*_1_, *M*_2_ and *M*_3_ polymerize between the TCu plates. Three flat capacitors are obtained (Figure 6), which are denoted with FCs. The configuration of the obtained membranes is shown in Figure 7.

### 3.3. Experimental Setup

The experimental setup used to study the magnetodielectric effects of the hMRE membranes is that shown in Figure 1 and consists of:

- A—neodymium permanent magnet, type VMM12-N54, generating the magnetic field of intensity *H*;
- B—RLC bridge, type ET7-20, from MNIPI (Republic of Belarus);
- Gs—Gaussmeter, Type DX-102;
- FC—working capacitor, fixed on the Hall probe through a spacing device (not represented in Figure 1) that has a micrometre screw;

By turning a screw (not shown in Figure 1), the distance between the permanent magnet and the plane capacitor determines the intensity of the magnetic field.

## 4. Conclusions

The hybrid magnetorheological elastomers (hMREs) can be successfully manufactured using a mixture of silicone rubber, silicone oil, carbonyl iron microparticles and graphene nanoparticles, absorbed in a cotton fabric. It is shown that the relative dielectric permittivity and electrical conductivity of hMREs increase with an increasing external magnetic field intensity and are significantly influenced by the amount of graphene used. The results obtained are due to the relaxation polarization of the silicone rubber, cumulated with that of the interfacial polarization of the graphene, due to the facilitation of the electron transport by the graphene and due to the magnetoconstriction of the hMREs when a magnetic field is applied.

## Figures and Tables

**Table 1:** Sample compositions.

*S_i_*	SR(g)	SO(g)	CI(g^3^)	nGr(g)
*S*_1_	6.90	1.08	7.86	0.00
*S*_2_	5.75	1.08	7.86	1.13
*S*_3_	4.60	1.08	7.86	2.26

**Table 2:** Membrane volume fractions ^1^.

*M_i_*	$\Phi_{SR}(\mathrm{wt}\%)$	$\Phi_{SO}(\mathrm{wt}\%)$	$\Phi_{CI}(\mathrm{wt}\%)$	$\Phi_{nGr}(\mathrm{wt}\%)$
*M*_1_	43.60	6.82	49.68	0.00
*M*_2_	36.35	6.82	49.68	7.15
*M*_3_	30.60	6.82	49.68	14.30

^1^$\Phi_{SR}$ (wt%), $\Phi_{SO}$(wt%), $\Phi_{CI}$(wt%) and $\Phi_{nGr}$(wt%) are the mass fractions of silicone rubber, silicone oil, carbonyl iron microparticles and graphene nanoparticles.

## Abbreviations

MRE	Magnetorheological elastomer
hMRE	Hybrid magnetorheological elastomer
FC	Flat capacitor
SR	Silicone rubber
CI	Carbonyl iron
nGr	Graphene nanoparticles
FT	Gauze bandage
TCu	Copper-plated textolite plates
